# Health benefit of vegetable/fruit juice-based diet: Role of microbiome

**DOI:** 10.1038/s41598-017-02200-6

**Published:** 2017-05-19

**Authors:** Susanne M. Henning, Jieping Yang, Paul Shao, Ru-Po Lee, Jianjun Huang, Austin Ly, Mark Hsu, Qing-Yi Lu, Gail Thames, David Heber, Zhaoping Li

**Affiliations:** Center for Human Nutrition, David Geffen School of Medicine, Department of Medicine, University of California Los Angeles, California, CA USA

## Abstract

The gut microbiota is an important contributor to human health. Vegetable/fruit juices provide polyphenols, oligosaccharides, fiber and nitrate (beet juice), which may induce a prebiotic-like effect. Juice-based diets are becoming popular. However, there is a lack of scientific evidence of their health benefits. It was our hypothesis that changes in the intestinal microbiota induced by a juice-based diet play an important role in their health benefits. Twenty healthy adults consumed only vegetable/fruit juices for 3 days followed by 14 days of customary diet. On day 4 we observed a significant decrease in weight and body mass index (p = 2.0E^−05^), which was maintained until day 17 (p = 3.0E^−04^). On day 4 the proportion of the phylum Firmicutes and Proteobacteria in stool was significantly decreased and Bacteroidetes and Cyanobacteria was increased compared to baseline and was partially reversed on day 17. On day 4 plasma and urine nitric oxide was increased by 244 ± 89% and 450 ± 360%, respectively, and urinary lipid peroxidation marker malondialdehyde was decreased by 32 ± 21% compared to baseline. General well-being score was increased at the end of the study. In summary a 3-day juice-based diet altered the intestinal microbiota associated with weight loss, increase in the vasodilator NO, and decrease in lipid oxidation.

## Introduction

Vegetable/fruit juice-based diets have been very popular recently. However, well designed controlled research studies with clinical outcome measures providing scientific evidence of potential health benefits of juice only diets are limited^1^. The consumption of vegetable/fruit juice during the abstinence from food provides essential nutrients and improves compliance.

Fruit and vegetables are rich sources of several biologically active components that contribute to general health and decrease the risk of chronic diseases such as cardiovascular disease^2^. They are the most ubiquitous source of phenolic compounds^3^. Polyphenols exert a variety of physiological effects *in vitro* including antioxidative, immunomodulatory and antimicrobial activities^4^.

The absorption of polyphenols in the small intestine is limited and considerable amounts of these polyphenols can be found in the colon. There the colonic bacteria metabolize polyphenols to smaller compounds, which in turn alter the abundance of bacteria in the intestinal microbiome. In addition, fruit and vegetable are rich in fermentable fiber with prebiotic activity. High fiber intake is associated with decreased risk of cardiovascular disease, type 2 diabetes and some forms of cancer^5^. Fiber is composed of oligosaccharides, which resist digestion in the small intestine and are transported to the colon where they provide energy for gut bacteria^6^. Growing evidence is demonstrating the role of the microbiota in the health benefits of dietary fiber consumption^6^.

In the human intestine the gut microbiota is an important contributor to human health and has been implicated in the development of obesity and obesity-related diseases such as diabetes and cardiovascular disease^7–9^. The two most abundant bacterial phyla in humans and in mice are Firmicutes (40–60%) and Bacteroidetes (20–40%) with lower abundance of Actinobacteria, Fusobacteria, Proteobacteria and Verrucomicrobia^10^. Recent studies show that dietary interventions with polyphenol rich extracts and foods, including dealcoholized red wine polyphenols, cocoa-derived flavanols, quercetin and grape anthocyanins, modulate the human gut microbiota by decreasing the abundance of Firmicutes and increasing Bifidobacteria, Lactobacillus and Verrucomicrobia^11–13^, which is also a key difference in the gut microbiota found in obese and lean individuals^14, 15^.

We therefore investigated whether the consumption of fruit and vegetable juices (6 bottles daily of mixtures of greens, roots, citrus, lemon, cayenne and vanilla almond) as part of a 3-day juice only program alters the intestinal microbiota in twenty healthy participants. Secondary outcomes of the study were to determine the effect of the 3-day juice based diet on change in weight and body composition and biomarkers of oxidation (urine malondialdehyde) and vasodilation (plasma and urine nitric oxide).

## Results

Thirty participants were screened. Twenty-five participants met enrollment criteria and were randomized and completed the 31-day study. Five participants were not able to provide stool samples at either day 4 or 17 and were excluded. All data included in this manuscript includes the twenty participants only (Table 1).

**Table 1:** Baseline characteristics of the study participants.

Variable	
Study Participants	
Screened (﻿N)	30
Enrolled/randomized (N)	25
Participants with all stool samples (N)	20
Age, years	32 ± 8
Gender	
Male (N)	5
Female (N)	15
Race	
African American (N)	1
Caucasian (N)	9
Asian ﻿(N)	8
Bi-Racial (N)	2
Height, cm	167 ± 5
Weight, kg	71 ± 18
BMI, kg/m^2^	25.5 ± 5

### Body weight and composition

**Figure 1:**
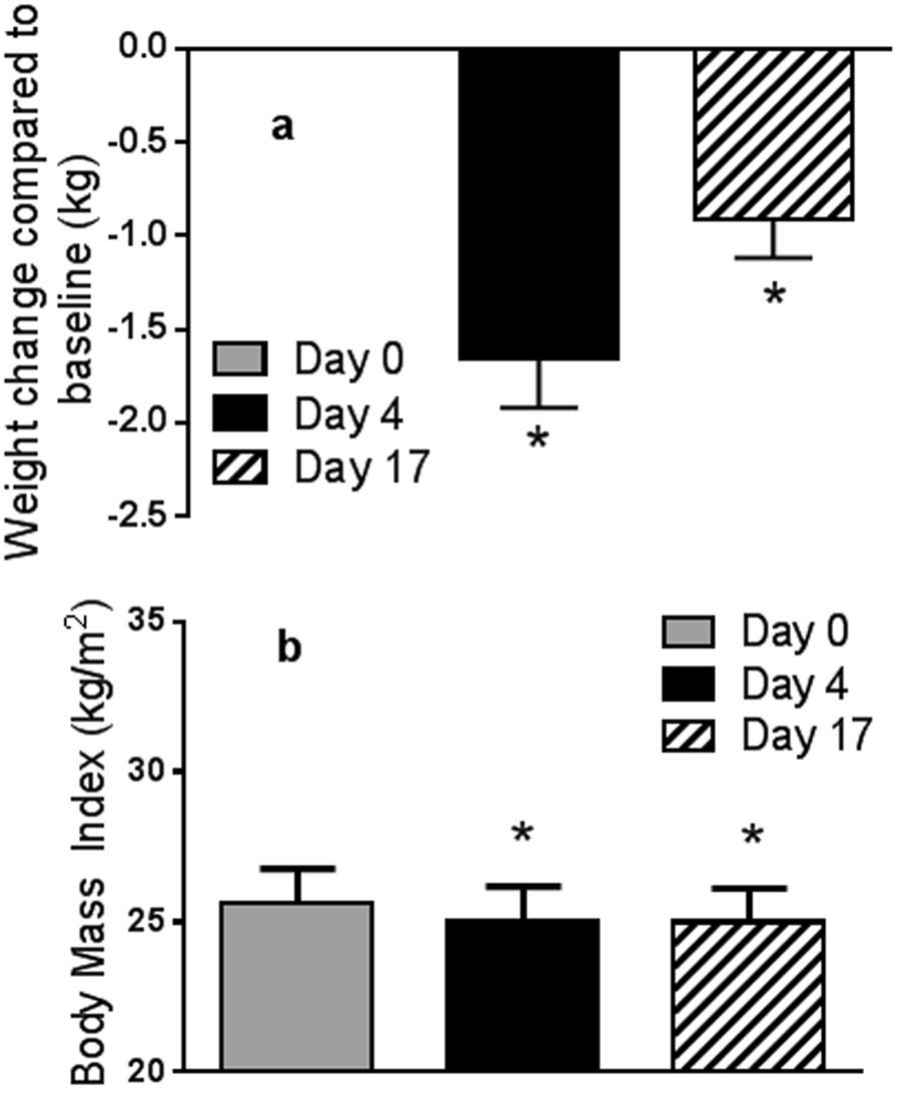
Effect of juice based diet on body weight (**a**) and body mass index (**b**). Data are mean ± SEM, n = 20. One way repeat measures ANOVA was performed. Difference to baseline is indicated; *p < 0.05.

According to the calorie content provided by the manufacturer the total calorie intake per person per day was 1310 kcal. During the 3-day juice intervention a significant weight loss of 1.7 ± 1.2 kg was observed (p = 2.0E^−05^) (Fig. 1a). After the 2-week follow up period body weight remained decreased (0.91 ± 0.9 kg) compared to baseline weight. Body mass index (BMI) was decreased by 0.6 ± 0.4 after the 3-day juice fast and remained decreased by 0.33 ± 0.3 after the 2 week follow up period (Fig. 1b).

### Intestinal microbiome

**Figure 2:**
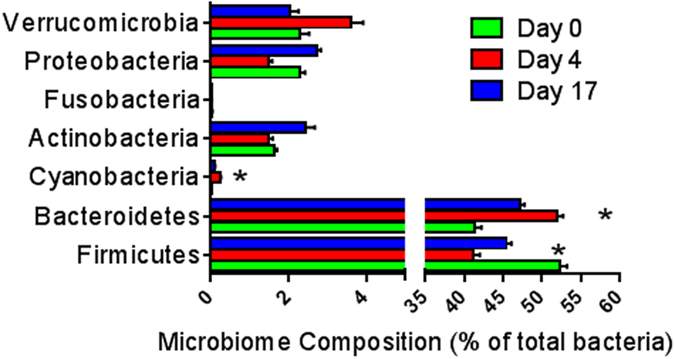
Effect of juice based diet on fecal microbiota (phylum) comparing baseline (day 0), after the juice intervention (day 4) and after two weeks of customary diet (day 17). Values are mean ± SEM (n = 20). Difference to baseline is indicated by *p < 0.05.

Fecal microbiota composition of 20 participants collected at day 0, 4 and 17 was analyzed by sequencing the V4 region of the 16S rDNA gene. Rarefaction curves for bacterial DNA sequences for each stool sample approached a plateau, indicating that sequence coverage was sufficient to encompass the majority of diversity contained within each sample (results not shown). The distribution of 16S rDNA genes at the phylum level was composed of Firmicutes > Bacteroidetes > Proteobacteria = Verrucomicrobia > Actinobacteria (52 ± 4, 41 ± 4, 2.3 ± 1, 2.3 ± 1 and 5 ± 4%, respectively). The juice consumption, however, was associated with a significant decrease in the proportion of the bacterial phylum Firmicutes (p = 0.014) and increase in Bacteroidetes (p = 0.026) and Cyanobacteria (0.003) at day 4 compared to baseline and was partially reversed to baseline proportions at day 17 (Fig. 2). The proportions of Verrucomicrobia, Proteobacteria, Actinobateria and Fusobacteria were not changed significantly (Fig. 2). Linear regression analysis of data from this study showed a significant positive correlation with day 4 body weight and Firmicutes proportion (p = 0.006), and a negative correlation with Bacteroidetes (p = 0.011) (Supplementary Fig. S1). There was no difference in indices of fecal community richness and diversity (α-diversity indices) throughout the intervention period (Supplementary Fig. 2; p = 0.49).

On the genus level the most prominent bacterium was *Bacteroides*, which was increased significantly at day 4 compared to baseline (Table 2 and Supplementary Fig. S3). Twenty different *Bacteroides* species were identified and the abundance of eight of them (*B. acidifaciens, B. caccae, B. fragilis, B. massiliensis, B. nordii, B. salyersiae, B. thetaiotaomicron, B. uniformis*) was increased on day 4 compared to baseline (Supplementary Fig. S3).

On the genus level the following bacterial populations were also significantly increased at day 4 compared to baseline (percent of baseline): *Halospirulina* (1467%), *Paraprevotella* (348%), *Barnesiella* (200%), *Odoribacter* (200%) and *Bacteroides* (144%) (Table 2). On the other hand, proportions of the following bacterial genera were decreased significantly at day 4 compared to baseline (percent of baseline): *Streptococcus* (8%), *Subdoligranulum* (30%), *Eisenbergiella* (40%), *Ruminiclostridium* (50%), and *Dialister* (67%) (Table 2). Except for *Streptococcus*, which was still significantly decreased to 19% of baseline at day 17, the proportions of all other bacterial genera were changed back to baseline values (Table 2).

**Table 2:** Effect of juice based diet on fecal microbiota (genus).

Genus	Day 0	Day 7	Day 17
*Anaeroarcus*	0.2 ± 0.03^b^	0.3 ± 0.05^a^	0.1 ± 0.02^b^
*Bacteroides*	25.6 ± 3.4^b^	37.0 ± 3.5^a^	30.2 ± 2.7^a,b^
*Barnesiella*	0.4 ± 0.15^b^	0.8 ± 0.3^a^	0.6 ± 0.2^a,b^
*Bilophila*	0.6 ± 0.3^a,b^	0.5 ± 0.2^b^	1.2 ± 0.4^a^
*Butyricimonas*	0.1 ± 0.05^a,b^	0.2 ± 0.07^a^	0.1 ± 0.03^b^
*Dialister*	3.6 ± 1.19^a^	2.4 ± 0.9^b^	3.6 ± 1.5^a,b^
*Eisenbergiella*	0.2 ± 0.06^a^	0.1 ± 0.01^b^	0.2 ± 0.05^a^
*Erysipelatoclostridium*	0.2 ± 0.04^a,b^	0.3 ± 0.11^a^	0.1 ± 0.02^b^
*Faecalibacterium*	6.7 ± 1.9^a,b^	4.2 ± 1.02^b^	7.5 ± 1.4^a^
*Haemophilus*	0.02 ± 0.008^a^	0.002 ± 0.001^b^	0.007 ± 0.004^a,b^
*Halospirulina*	0.02 ± 0.007^b^	0.2 ± 0.07^a^	0.09 ± 0.07^b^
*Odoribacter*	0.3 ± 0.11^b^	0.6 ± 0.1^a^	0.4 ± 0.09^b^
*Oscillospira*	2.3 ± 1.0^a^	2.1 ± 0.5^b^	2.6 ± 0.84^a,b^
*Parabacteroides*	2.5 ± 0.66^a^	2.4 ± 0.52^a,b^	3.3 ± 0.6^b^
*Paraprevotella*	0.2 ± 0.12^b^	0.8 ± 0.4^a^	0.8 ± 0.38^a,b^
*Ralstonia*	0.002 ± 0.000^a^	0.001 + 0.000^b^	0.001 ± 0.000^a,b^
*Ruminiclostridium*	1.0 ± 0.28^a^	0.5 ± 0.15^b^	0.5 ± 0.12^a,b^
*Streptococcus*	1.5 + 0.63^a^	0.1 + 0.03^b^	0.3 + 0.09^b^
*Subdoligranulum*	4.1 ± 1.16^a^	1.5 ± 0.34^b^	2.5 ± 0.7^a,b^

This table includes only significantly altered bacteria. Supplementary Table S2 includes bacteria that did not change significantly. Data presents proportions of bacteria as percent of total count. Values are mean ± SEMs (n = 20). ^a,b^Labeled means without a common superscript letter differ, *p* < 0.05.

The functional analysis of the microbial metagenome using PICRUST showed no difference in bacterial nitrogen metabolism (Supplementary Fig. S4).

### Plasma antioxidant capacity and urine lipid peroxidation

**Figure 3:**
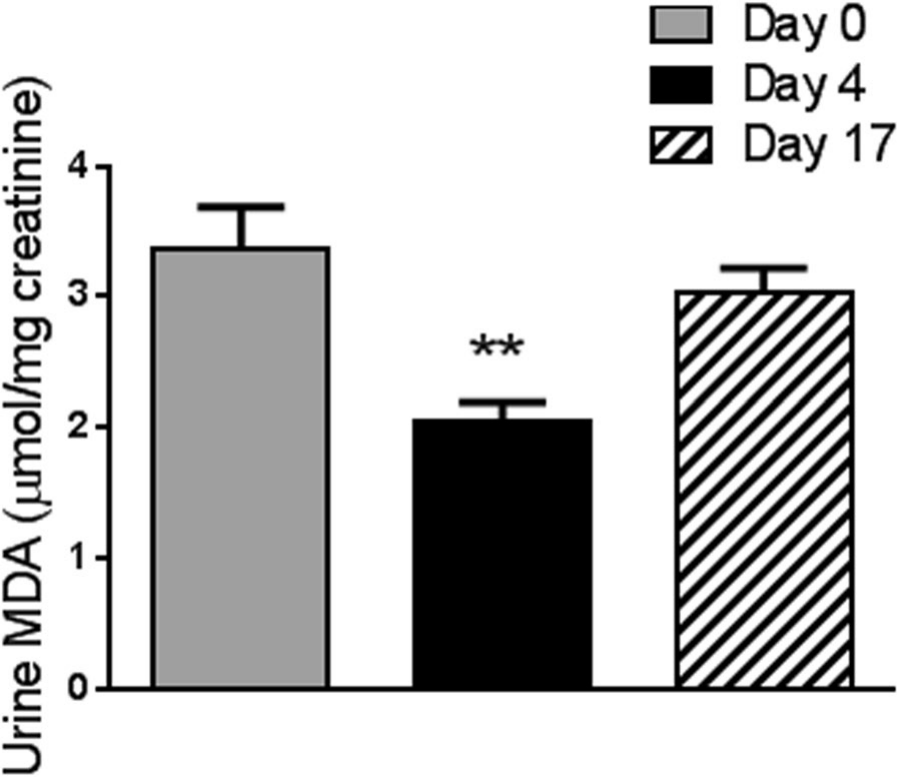
Effect of juice based diet on urinary malondialdehyde concentration. Data are mean ± SEM. One way repeat measures ANOVA was performed. The difference to baseline is indicated **p < 0.001.

The effect of juice based diet on plasma antioxidant capacity was determined by analyzing the trolox equivalents (TE) using the TEAC method. There was no change in plasma TE comparing samples collected at day 4 and 17 with baseline samples (data not presented). Lipid peroxidation was determined by the analysis of urine malondialdehyde (MDA). Urine MDA was significantly decreased by 40% in 6-hour urine collected at day 4 compared to baseline urine (p = 0.01) and returned to baseline levels at day 17 (Fig. 3).

### Plasma and urine nitric oxide

**Figure 4:**
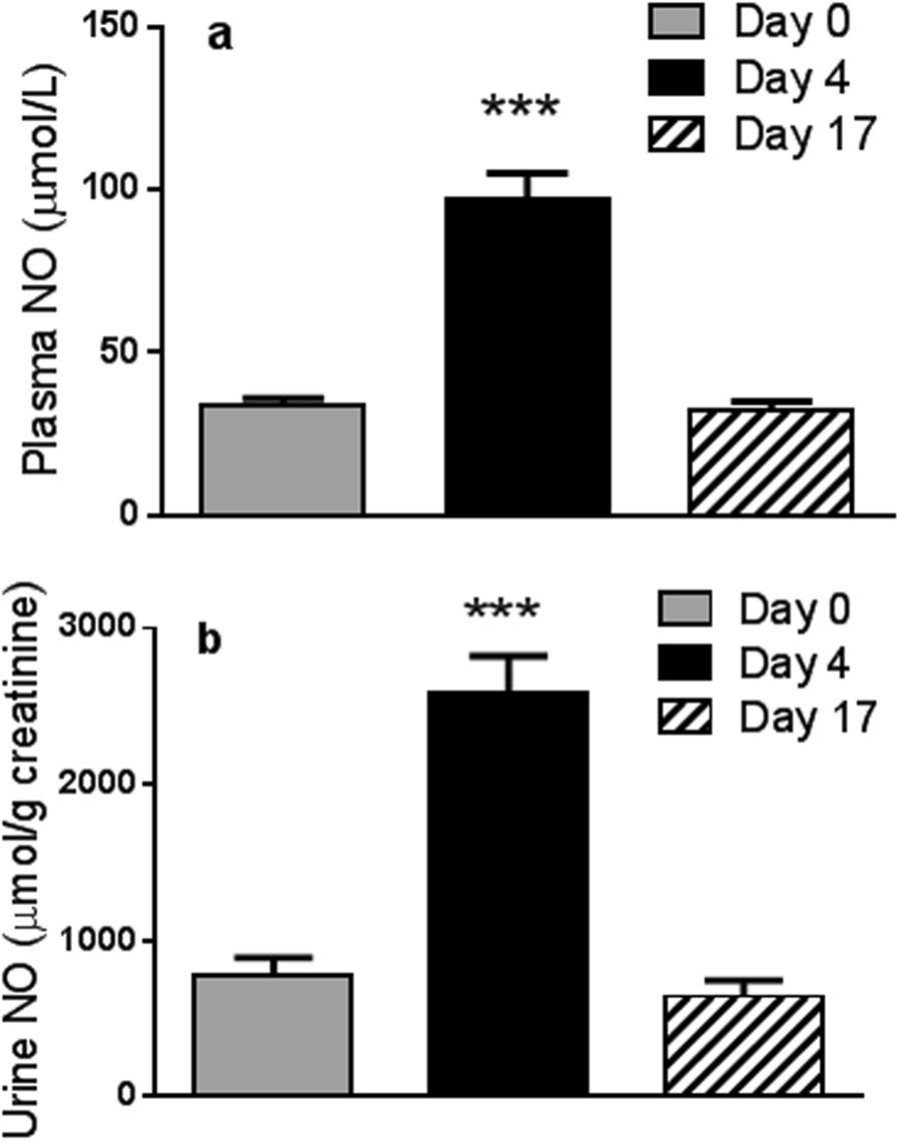
The effect of juice based diet on (**a**). Plasma nitric oxide and (**b**). Urinary nitric oxide. Data are mean ± SEM (n = 20). One way repeat measures ANOVA was performed. The difference to baseline is indicated ***p < 1.0E^−08^.

To evaluate potential effects of vegetable and fruit nitrate content on vasodilation we determined the effect of juice based diet on plasma and urine nitric oxide (NO) concentrations. Both plasma and urine NO were significantly increased 3-fold for plasma and 5-fold for urine at 4 days compared to baseline (p = 1.0E^−06^) (Fig. 4). All NO values returned to baseline concentrations at 17 days **(**Fig. 4).

### General well-being score

**Figure 5:**
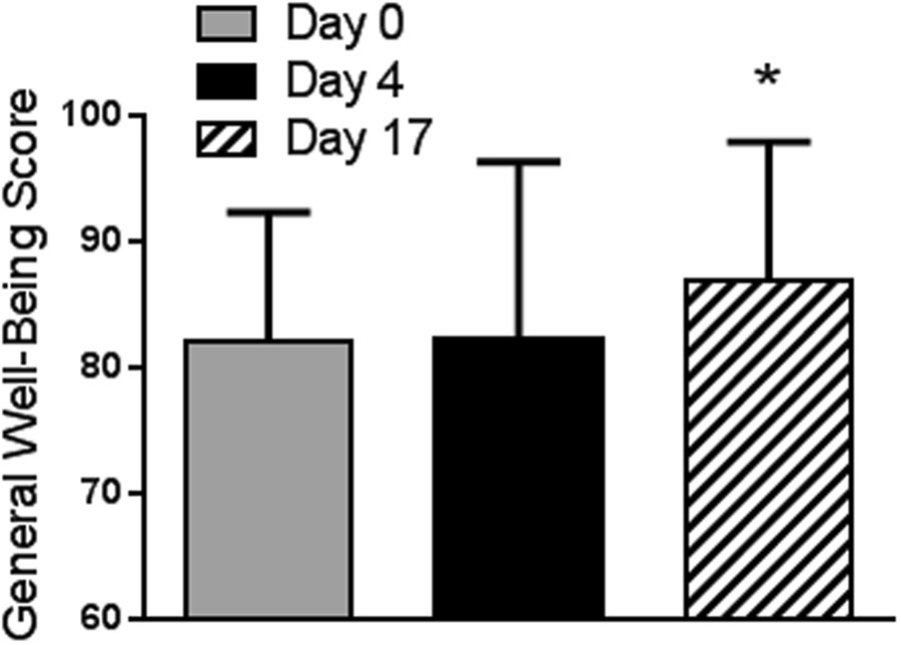
Effect of juice based diet on Wellness Score. Data are mean ± SEM (n = 20). One way repeat measures ANOVA was performed. The difference to baseline is indicated *p < 0.05.

All participants completed a general well-being questionnaire at baseline, day 4 and at the end of study. There was no difference in well-being score after the 3-day juice period (82.4 ± 14) compared to baseline (82.2 ± 10). However, at the end of the study the well-being score (87 ± 11) was significantly increased (p = 0.006) (Fig. 5).

## Discussion

The scientific base for the popularity of juice based diets has not been explored. Here we demonstrated that a 3-day juice-based diet of drinking 6 bottles of fruit/vegetable juice blends (16 oz ea) resulted in a significant decrease in body weight (p = 2.0E^−05^). The observed weight loss remained significant and persisted over the following 2 weeks and may be related to changes in the microbiome (p = 0.003).

The two most abundant bacterial phyla in humans are Firmicutes (40–60%) and Bacteroidetes (20–40%)^10^. Relative proportional abundance of Firmicutes has been associated with increased body weight and that of Bacteroidetes with low body weight. The 3-day juice based diet of only drinking 6 bottles of fruit/vegetable juice blends induced significant changes in the intestinal microbiota. The proportional abundance of the phylum Firmicutes was significantly decreased, while Bacteroidetes was significantly increased when comparing end of juice based diet (day 4) to baseline (Table 1). Human *Bacteroides* species are able to degrade diverse plant fiber and complex polysaccharides, including pectin and xylans from fruit and vegetable^16, 17^. *Bacteroides ovatus, B. thetaiotaomicron and B. uniformis* ferment a particularly wide range of complex polysaccharides^16^. In the present study eight *Bacteroides* species were significantly increased after the juice based diet. A significant increase in the proportion of these *Bacteroides* species (*B. ovatus, B. acidifaciens and B. xylanisolvens*) was also observed in another human intervention study in individuals with metabolic syndrome who included the prebiotic resistant starch in their diet^18^. In that study the increase in *Bacteroides* was associated with a decrease in markers of metabolic syndrome (cholesterol, blood pressure, etc.). In another study *B. thetaiotaomicron* was used as probiotic in combination with a prebiotic in rats fed a high fat diet, resulting in a significant decrease in weight and postprandial plasma triglyceride levels^19^. Products of fiber/complex carbohydrate metabolism are short chain fatty acids, which have been shown to play an important role in the cardiovascular health benefits of fiber consumption^20^.

The only other human study investigating the effect of juice based diet on changes in the gut microbiota was published by Remely *et al*.^21^. They observed changes in the stool microbiome in participants following a traditional program in an Austrian Monastery including a small amount of cereal, vegetable, fruit and herbal tea^21^. After one week of the program an increase in microbial diversity was observed as well as the abundance of *Akkermansia* and *Bifidobacteria* was increased and the abundance of *Enterobacteria* and *Lactobacilli* was decreased as determined by quantitative PCR of 16S rDNA of the bacteria of interest^21^.

In the present study the consumption of the juices was associated with a significant increase in plasma and urine nitric oxide. Nitric oxide (NO), a known vasodilator, is an important factor in cardiovascular disease. Impaired eNOS activity and lack of bioavailable NO play a role in the development and progression of endothelial dysfunction that leads to arterial stiffness and an increase in blood pressure^22^. A study by Velmurugan *et al*. demonstrated that the consumption of dietary nitrate from beetroot juice resulted in significant increase in ultrasound flow-mediated dilatation in hypercholesterolemic patients^23^. Dietary interventions leading to increased blood NO concentrations may be beneficial in the prevention of heart disease.

Sources of nitric oxide (NO) are either the endogenous formation through the endothelial arginine nitric oxide synthase (eNOS) pathway or from dietary sources in form of nitrate^24^. One of the juices consumed during the juice fast was prepared from green leafy vegetable and one from beetroot, which are both good dietary sources of nitrate^24^. Polyphenols have been shown to enhance eNOS activity^22, 24^. Due to the high polyphenol content of the fruit/vegetable juices stimulation of endogenous eNOS activity may have contributed to the increase in NO. Endothelial dysfunction is one of the hallmark warning signs of cardiovascular disease and is involved in disease states such as atherosclerosis and hypertension, in which the normal function of endothelium is severely affected^25^. Dietary nitrate is mostly reduced to nitrite by nitrate-reducing bacteria in the oral cavity but also by bacteria in the lower intestinal tract^24, 26, 27^. In the present study, however, our functional analysis of the microbial metagenome using PICRUST showed no difference in the nitrogen metabolism (p = 0.24).

We observed a decrease in the concentration of urinary lipid peroxidation product malondialdehyde (MDA) after the 3-day juice fast. This may have been based on the high polyphenol content providing antioxidant protection for lipids in the intestine during the digestion. Similar effects on postprandial lipid peroxidation were observed by our laboratory and others utilizing other polyphenol sources. For example the addition of a spice mix or red wine to a high fat meal dramatically decreased postprandial urinary MDA formation^28, 29^. Another study by Bub *et al*. also supports the antioxidant effect of polyphenol rich fruit juices^30^. In this randomized, crossover-design study in healthy men, a daily consumption of polyphenol-rich juices (330 ml/d) consumed for 2 weeks decreased plasma malondialdehyde compared to baseline^30^. In addition, urine MDA may have been decreased based on the low fat content of the juice fast (15% calories from fat).

The vegetable/fruit juice based diet consumption showed a delayed response to improved well-being after participants returned to their regular diet for two weeks. Since the microbiota changes mostly returned to baseline levels, it appears unlikely that changes in the well-being were related to the gut microbiota. As mediator of mood, appetite and sleep, serum levels of the monoamine neurotransmitter serotonin could play a role in well-being. However, no significant change in serum serotonin levels were found (data not shown). Possibly participants felt good about the weight loss that was maintained through the two weeks after returning to the customary diet.

In summary the 3-day vegetable/fruit juice-based diet induced significant changes in the intestinal microbiota which were associated with weight loss. Further mechanistic studies are required to confirm that changes in the microbiota are directly linked to weight loss. The juice- based diet also, significantly increased serum and urine NO and decreased a marker of lipid oxidation. Future studies will need to be performed to determine if these biochemical changes are associated with vasodilation and improved cardiovascular health.

## Methods

### Study design and juice intervention

The study was carried out in accordance with the guidelines of the Office for Protection of Research Subjects of the University of California, Los Angeles. The clinical protocol was approved by the internal review board (IRB) of the University of California, Los Angeles. The study was registered at the NIH Clinical Trial Registry: NCT02377063 on 2/18/2015.

All subjects provided written informed consent before the study began. The study was divided into three periods: 2-week run-in period, 3-day juice fast and 2-week follow-up period. Healthy subjects who consume <3 servings of fruits/vegetables were recruited to the study. During the run-in period the participants were asked to continue their usual diet and refrain from consuming vitamins and antibiotics. During the intervention period all participants only consumed 6 bottles of vegetable/fruit juice daily for 3 days. The 6 different types of vegetable/fruit blends were prepared from the following fruits and vegetables: Green mixes were blended from apple, cucumber, celery, romaine lettuce, lemon and limited amount (<2%) of spinach, kale and parsley. One of the green mixes also contained ginger. The root mix was a juice blend of apple, lemon, ginger and beet. The citrus mix contained apple, pineapple, and very limited amount (<1%) of lemon and mint. Lemon cayenne water consisted of filtered water with cayenne and lemon and “vanilla almond (VA)” was a blend of almond, dates, sea salt and vanilla bean. The nutritional facts are listed in Supplementary Table S1. Each bottle contained 16 oz. or 2 servings. The total caloric content of all 6 bottles providing 1310 kcal per day. The juices provided 38 g of total fat per day (266 kcal, 20% calories from fat) and 28 g of fiber.

During the follow-up period participants resumed their usual diet consumed during the run-in period following the same fruit and vegetable serving, vitamin and antibiotic restrictions. Juices were obtained from Pressed Juicery (Santa Monica, CA). Nutrition facts are provided in Supplementary Table S1. On day 0, 4 and 17 participants completed a Psychological General Well Being Questionnaire (http://www.opapc.com).

### Subjects

Twenty-five healthy women and men 18–50 years of age with a custom diet including <3 servings of fruits/vegetables per day were recruited through local advertisement. Subjects with a history of diabetes mellitus on medications, hyperlipidemia on medications, or other serious medical condition within 6 months prior to screening were excluded. In addition, individuals using antibiotics or laxatives during 2 months prior to the study were excluded.

### Blood and urine collection

#### Fasting

EDTA blood samples and 24 hr urine samples were collected at baseline, day 4 and day 17. Blood was centrifuged 1500 × g for 10 min at 10 °C and plasma stored at 80 °C until analysis.

### Stool Collection

A total of three stool samples were collected from each subject: at baseline, day 4 and day 17. Each time an aliquot of the stool specimen was collected by the participant and delivered to the UCLA Center for Human Nutrition in a cooler within a few hours of collection. At the laboratory stool was aliquoted into smaller vials and frozen immediately and stored at −80 °C.

### Bacterial DNA Sequencing

DNA from stool was extracted using the MoBio power soil DNA isolation kit (MoBio Laboratories, Inc., Carlsbad, CA). The quality and quantity of the DNA was confirmed using a Nanodrop 1000 (Thermo Fisher Scientific, Wilmington, DE). The 16S rRNA gene V4 variable region PCR primers 530/926 with barcode on the forward primer were used in a 30 cycle PCR using the HotStarTaq Plus Master Mix Kit (Qiagen, USA) under the following conditions: 94 °C for 3 min, followed by 28 cycles of 94 °C for 30 s, 53 °C for 40 s and 72 °C for 1 min, after which a final elongation step at 72 °C for 5 min was performed. After amplification, PCR products are checked in 2% agarose gel to determine the success of amplification and the relative intensity of bands. Sequencing was performed at MR DNA (www.mrdnalab.com, Shallowater, TX, USA) on a MiSeq (Illumina, San Diego, CA) following the manufacturer’s guidelines. Sequence data were processed using a proprietary analysis pipeline (MR DNA, Shallowater, TX, USA). Operational taxonomic units (OTUs) were defined by clustering at 3% divergence (97% similarity). Final OTUs were taxonomically classified using BLASTn against a curated GreenGenes database^31^. Within community diversity (α-diversity) was calculated using Quantitative Insights Into Microbial Ecology (QIIME) software package^32^. Analysis of α-diversity (Shannon index) was performed by a one-way ANOVA. β-diversity was measured by calculating the weighted UniFrac distances^33^ using QIIME default scripts, and weighted UniFrac PCoA biplot was visualized using *EMPeror*. Statistical difference between different time points was analyzed by PERMDISP. Microbiome data was further analyzed using PICRUST (phylogenetic investigation of communities by reconstruction of unobserved states) to predict the functional composition of the microbial community’s metagenome from its 16S profile by using marker gene data and a database of reference genomes^34^.

### Body Composition

Body composition was measured using the Tanita-BC418 bioelectrical impedance analyzer (Tanita Corp., Japan).

### Malondialdehyde

Urine malondialdehyde concentration was determined in form of thiobarbituric acid (TBA) reactive substances by deproteinization and derivatization with TBA according to the method of Korchazhkina *et al*.^35^. In brief, 0.6 mL urine was mixed with 0.4 mL of water and 3 mL of H3PO4 (1%V/V) and vortexed. One ml of TBA solution was added and incubated in a 100 °C water bath for 60 min. Samples were cooled on ice and centrifuged at 18,000 × g for 10 min. An aliquot of 50 µL supernatant was injected into the HPLC system for analysis. Chromatographic determinations were performed on a Waters 2690 HPLC (Waters, Milford, MA) equipped with a Waters 474 fluorescence detector (Waters, Milford, MA) at Ex (ex-citation) 515 nm and Em (emission) 550. An Agilent Zorbax SB C18 reversed-phase column (150 mm, 4.6-mm inside diameter; 3.5-µm particles; Waters, Milford, MA) was used for separation at an ambient temperature. A linear gradient from 10% A and 90% B to 60% A and 40% B over 20 min (A: acetonitrile and B: 0.1% phosphoric acid in water) at a flow rate of 0.8 mL/min was used for the elution. The reagent 1,1,3,3-tetramethoxypropane was used to prepare a standard curve.

### Nitric oxide

Nitric oxide (NO) is oxidized in the body to the metabolites nitrate (NO^3−^) and nitrite (NO^2−^), which are excreted in the urine. Nitrate/nitrite concentrations were measured in the form of NO in urine and serum samples by an ozone chemiluminescence method (Sievers Instruments, Boulder, CO). Serum samples were mixed with ethanol in a ratio of 1:2 and stored at 4 °C overnight. After centrifugation for 20 min at 17 000 × g an aliquot of the supernatant was injected into the reaction chamber of the NO analyzer. Urine was centrifuged at the same speed and injected. Total nitrate and nitrite were analyzed by injecting 5 μL of each microdialysate sample into a purge vessel containing a solution of vanadium (III) chloride (50 mmol/L) in hydrochloric acid (1 mol/L) at 95 °C, continuously purged with a stream of nitrogen gas, connected to a Sievers 280i Nitric Oxide Analyser (GE Analytical Instruments, Boulder, CO, USA). The concentration was determined in comparison to a sodium nitrate standard calibration curve.

### Urine creatinine

The urinary creatinine concentration was measured by using a Stanbio Direct Creatinine LiquiColor Kit (Stanbio LaboratoryBoerne, TX, USA) according to manufacturer’s instructions. A creatinine standard calibration curve was generated by plotting the absorbance measured at 510 nm of a series concentration of creatinine standard solutions reacting with working reagent provided by the kit. The urinary creatinine level was measured in the same manner after a proper dilution with distilled water.

### Statistics

Statistical analyses were performed using IBM SPSS Statistics version 22 (IBM Corporation, Armonk, NY, USA). Data were evaluated with One-way repeated measures ANOVA with a Bonferroni’s post-test if the assumption are met, and those without were analyzed using non-parametric test (Friedman test with a Dunn’s post-test). *P*-values < 0.05 were considered statistically significant. Correlation was evaluated using GraphPad Prism 6 (La Jolla, CA).

## Electronic supplementary material

Supplementary Information
