# Supplementary material for: Health benefit of vegetable/fruit juice-based diet: Role of microbiome
Source: Sci Rep. 2017 May 19;7:2167. doi: 10.1038/s41598-017-02200-6 (PMC5438379; doi:10.1038/s41598-017-02200-6)
Supplement: Supplementary file 1 — Supplementary Information [file 41598_2017_2200_MOESM1_ESM.docx]

Health benefit of vegetable/Fruit juice-based diet: Role of microbiome

Susanne M. Henning*, Jieping Yang, Paul Shao, Ru-Po Lee, Jianjun Huang, Austin Ly, Mark Hsu, Qing-Yi Lu, Gail Thames, David Heber, Zhaoping Li

Center for Human Nutrition, David Geffen School of Medicine, Department of Medicine, University of California Los Angeles, CA, USA

**Supplementary Information**

Supplementary Table S1. Juice composition.
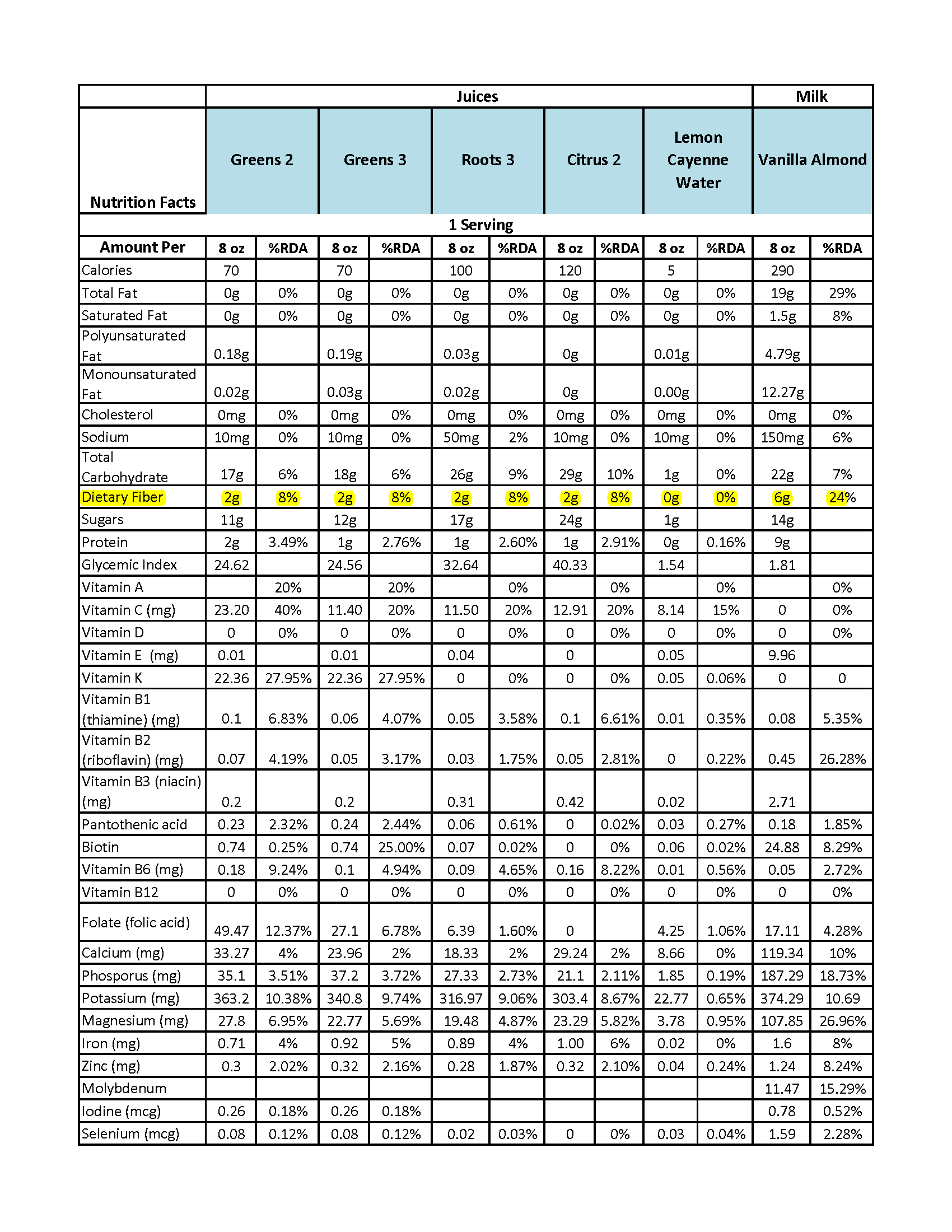


Supplementary Table S2. Effect of juice based diet on fecal microbiota (genus). This table is supplemental to Table 2 and includes bacteria not changed significantly.

| **Genus** | **Day 0** | **Day 7** | **Day 17** |
| --- | --- | --- | --- |
| *Acetivibrio* | 0.088 + 0.034 | 0.156 + 0.064 | 0.101 + 0.053 |
| *Acidaminococcus* | 0.051 + 0.023 | 0.143 + 0.119 | 0.080 + 0.041 |
| *Akkermansia* | 2.288 + 1.070 | 3.594 + 1.473 | 2.048 + 0.949 |
| *Bifidobacterium* | 1.089 + 0.363 | 0.815 + 0.379 | 2.026 + 1.113 |
| *Blautia* | 5.094 + 1.152 | 3.644 + 0.520 | 3.473 + 0.633 |
| *Catenibacterium* | 0.326 + 0.290 | 0.191 + 0.178 | 0.389 + 0.278 |
| *Clostridium* | 5.190 + 0.720 | 5.490 + 0.865 | 4.708 + 0.563 |
| *Collinsella* | 0.406 + 0.111 | 0.555 + 0.235 | 0.307 + 0.086 |
| *Coprococcus* | 0.572 + 0.171 | 0.298 + 0.056 | 0.396 + 0.105 |
| *Desulfovibrio* | 0.097 + 0.052 | 0.065 + 0.024 | 0.178 + 0.091 |
| *Dorea* | 0.789 + 0.233 | 0.532 + 0.095 | 0.419 + 0.101 |
| *Dysgonomonas* | 0.018 + 0.009 | 0.186 + 0.176 | 0.011 + 0.002 |
| *Eubacterium* | 8.526 + 1.684 | 6.974 + 1.097 | 5.550 + 0.726 |
| *Flavonifractor* | 0.243 + 0.076 | 0.335 + 0.075 | 0.341 + 0.080 |
| *Fusicatenibacter* | 0.766 + 0.206 | 0.443 + 0.072 | 0.594 + 0.099 |
| *Gemmiger* | 1.654 + 0.472 | 1.217 + 0.303 | 1.132 + 0.237 |
| *Holdemanella* | 0.343 + 0.236 | 0.108 + 0.057 | 0.217 + 0.116 |
| *Lachnoclostridium* | 0.448 + 0.071 | 0.571 + 0.101 | 0.514 + 0.092 |
| *Lachnospira* | 0.281 + 0.101 | 0.659 + 0.462 | 0.267 + 0.136 |
| *Lactobacillus* | 0.299 + 0.119 | 0.238 + 0.070 | 0.181 + 0.064 |
| *Megamonas* | 1.891 + 1.825 | 0.787 + 0.546 | 0.585 + 0.421 |
| *Pantoea* | 0.196 + 0.176 | 0.005 + 0.001 | 0.18 + 0.008 |
| *Parasutterella* | 0.615 + 0.340 | 0.486 + 0.211 | 0.846 + 0.353 |
| *Phascolarctobacterium* | 0.707 + 0.267 | 1.483 + 0.462 | 1.940 + 0.627 |
| *Prevotella* | 6.452 + 3.578 | 4.001 + 2.210 | 4.247 + 1.886 |
| *Roseburia* | 1.241 + 0.234 | 0.751 + 0.139 | 1.061 + 0.228 |
| *Ruminococcus* | 2.457 + 0.743 | 4.411 + 1.732 | 3.535 + 1.381 |
| *Shigella* | 0.296 + 0.187 | 0.047 + 0.014 | 0.143 + 0.088 |
| *Sporobacter* | 0.092 + 0.039 | 0.108 + 0.046 | 0.116 + 0.030 |
| *Sutterella* | 0.368 + 0.175 | 0.306 + 0.166 | 0.252 + 0.117 |
| *Turicibacter* | 0.060 + 0.021 | 0.085 + 0.067 | 0.873 + 0.546 |
| *Tyzzerella* | 0.350 + 0.099 | 0.555 + 0.154 | 0.485 + 0.159 |
| *Veillonella* | 0.587 + 0.466 | 0.122 + 0.052 | 0.322 + 0.233 |

Values are means + SEMs (n = 20).

Supplementary Figure S1. Correlation of relative proportional abundance of Firmicutes and Bacteroidetes to body weight after 3-day of juice fast. Correlation was evaluated using GraphPad Prism6.

**R=0.35, p=0.006**

**R=0.31, p=0.011**

Supplementary Figure S2. There was no difference of within community diversity (α-diversity) between samples from day 0, day 4 and day 17 (p=0.49). α-diversity was calculated using Quantitative Insights Into Microbial Ecology (QIIME). N=20. Statistical difference was determined using a one-way ANOVA.


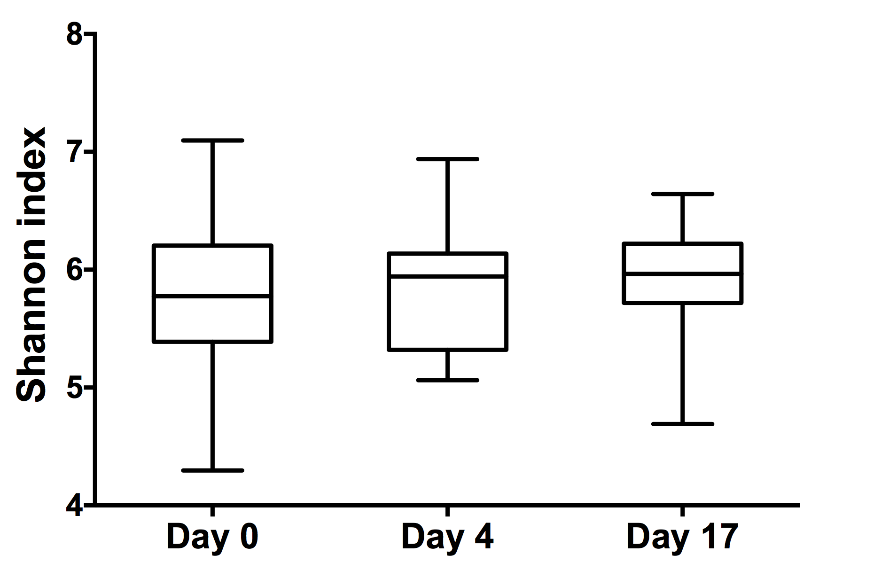


Supplementary Figure S3. Proportion of *Bacteroides* species of total bacteria present in stool samples.

Supplementary Figure S4. There was no significant difference in nitrogen metabolism of fecal bacteria between samples collected on day 0, day 4 and day 17 (p=0.29). The effect on nitrogen metabolism was determined using PICRUST program. N=20.
